# Central retinal artery occlusion – rethinking retinal survival time

**DOI:** 10.1186/s12886-018-0768-4

**Published:** 2018-04-18

**Authors:** Stephan Tobalem, James S. Schutz, Argyrios Chronopoulos

**Affiliations:** 1 Department of Ophthalmology, University Hospitals and School of Medicine, Geneva, Switzerland; 2 Department of Ophthalmology, Addenbrooke’s Hospital, Cambridge University Hospital NHS Foundation Trust, Box 41, Hills Road, Cambridge, CB2 0QQ UK

**Keywords:** Central retinal artery occlusion, Retinal infarction, Retinal stroke, Choroidal infarction, Post-operative visual loss, Cherry red spot

## Abstract

**Background:**

The critical time from onset of complete occlusion of the central retinal artery (CRA) to functionally significant inner retinal infarction represents a window of opportunity for treatment and also has medical-legal implications, particularly when central retinal artery occlusion (CRAO) complicates therapeutic interventions. Here, we review the evidence for time to infarction from complete CRAO and discuss the implications of our findings.

**Methods:**

A Medline search was performed using each of the terms “central retinal artery occlusion”, “retinal infarction”, “retinal ischemia”, and “cherry red spot” from 1970 to the present including articles in French and German. All retrieved references as well as their reference lists were screened for relevance. An Internet search using these terms was also performed to look for additional references.

**Results:**

We find that the experimental evidence showing that inner retinal infarction occurs after 90–240 min of total CRAO, which is the interval generally accepted in the medical literature and practice guidelines, is flawed in important ways. Moreover, the retinal ganglion cells, supplied by the CRA, are part of the central nervous system which undergoes infarction after non-perfusion of 12–15 min or less.

**Conclusions:**

Retinal infarction is most likely to occur after only 12–15 min of complete CRAO. This helps to explain why therapeutic maneuvers for CRAO are often ineffective. Nevertheless, many CRAOs are incomplete and may benefit from therapy after longer intervals. To try to avoid retinal infarcton from inadvertent ocular compression by a headrest during prone anesthesia, the eyes should be checked at intervals of less than 15′.

## Background

Central retinal artery occlusion (CRAO) of sufficient duration causes a stroke, infarction of the inner retina including the retinal ganglion cells and their axons which form the optic nerve, central nervous system tissue [1, 2]. The retinal ganglion cell layer is critically dependent on oxygen supplied by the end-artery circulation of the central retinal artery (CRA) [3, 4].

Retinal stroke from CRAO typically presents as acute, catastrophic, permanent visual loss in one eye, about 80% of the time with a visual acuity of 20/400 or worse, an afferent pupillary defect, a classic retinal cherry red spot on ophthalmoscopy from opacification of the infarcted ganglion cell layer surrounding the fovea, and sometimes a visible CRA embolus or other retinal arterial emboli [1, 3, 5]. Sometimes, segmentation of retinal blood columns (box-carring) with stagnation of retinal arterial flow may be present, indicating complete CRAO [1, 3]. Areas of inner retina supplied by cilioretinal arteries, which are common and not branches of the CRA, survive with visual function; if a cilioretinal artery supplies the macula despite surrounding infarction from CRAO, central vision may remain normal [4, 6]. Infarction of the retinal ganglion cell somata is followed by progressive axonal degeneration with pale optic atrophy evident after some weeks [3]. The outer retina including the photoreceptors and underlying retinal pigment epithelium is supplied with oxygen by the lush choroidal circulation derived from the long and short posterior ciliary arteries and is not infarcted in pure CRAO [3]. Nevertheless, conduction of visual information centrally is blocked by the loss of retinal ganglion cell function.

The critical time from onset of complete CRA occlusion to inner retinal infarction represents a window of opportunity for emergency treatment to try to prevent catastrophic visual loss. We examined the experimental evidence for the interval between complete CRA occlusion and subsequent retinal ganglion cell infarction. Therapeutic implications for emergency treatment as well as medical-legal considerations are discussed.

## Methods

Medline was systematically searched using the terms “retinal stroke”, “central retinal artery occlusion”, “retinal infarction”, “retinal ischemia”, and “cherry red spot” from 1970 to the present including articles in English, French, and German literature. These terms were connected with “or”. An Internet search using these search terms was also performed to look for additional references with no year restriction. All retrieved references as well as their reference lists were screened for evidence relevant to determination of the time to infarction of complete CRAO as well as articles relevant to the physiology of retinal stroke.

### Eligible criteria

All publications obtained from Internet-based searches were screened by the above predefined selection criteria. Eligible studies were all studies reporting on the time to retinal infarction from complete CRAO as well as articles relevant to the physiology of retinal stroke. Two reviewers (S.T. and S.J) completed the assessment of search results to identify included studies.

## Results

It is generally accepted that the human ganglion cell layer will survive without infarction and with preservation of vision if CRA circulation is restored after up to 90 to 240 min of complete non-perfusion from CRAO [3, 7–15]. We find that this clinically important time limit for inner retinal non-perfusion has been greatly overestimated for several reasons.

First, it is based on a series of experiments on non-human primates by Hayreh and coworkers [11, 12, 14] which are inappropriate for extrapolation to human CRAO because:

- The experiments were performed on barbiturate anesthetized monkeys. Barbiturate anesthesia has an important neuro-protective effect which prolongs neuronal survival during ischemia [16–20].
- The experiments were not controlled for the presence of hypothermia in the experimental animals which was likely to have had an additional neuro-protective effect prolonging retinal ganglion cell survival [17].
- Clamping of the CRA just before its entrance into the optic nerve sheath was performed in all the monkey experiments but is not an appropriate model for complete CRAO because there is collateral circulation to the CRA distal to the clamp in the monkey (and in the human eye) derived mostly from posterior ciliary artery branches of the ophthalmic artery [4, 21]. Persistent CRA perfusion was demonstrated in most of the experimental animals by fluorescein angiography with CRA filling in as little as 4 s; this was caused by either incomplete clamping of the CRA or collateral circulation distal to the clamping [11, 12]. Collateral circulation distal to the clamped CRA at its dural entry into the optic nerve sheath may very much delay or prevent inner retinal ischemia and infarction from CRAO; consequently, proper determination of the interval to inner retinal infarction after complete CRAO should be predicated on actual complete CRAO with no flow to the inner retina.
- While it has been suggested that the site of CRA occlusion in humans is at the dural entry of the CRA into the optic nerve [13, 22–24], careful review of the references cited as evidence do not support this assertion nor can we find substantiation for this anywhere else in the peer reviewed literature. CRAO sometimes occurs at the posterior surface of the lamina cribrosa [1, 25]. There is good clinical evidence that human CRA occlusion is often on the optic nerve head where the CRA narrows suddenly, branching into its major subdivisions, much more distal than the entrance of the CRA into the optic nerve dura [9]; the CRA at this point on the surface of the optic nerve head is normally an end-artery with no collateral circulation [4]. Complete CRAO with no flow through the CRA, whether embolic or thrombotic, is well documented and would not occur if occlusion were present at the entry of the CRA into the optic nerve dural sheath because there would be residual flow and filling of the CRA from variable fine arterial collaterals along the course of the CRA within the optic nerve and collaterals in the area of the scleral wall from the circle of Zinn-Haller [4, 26].

Second, the retinal ganglion cells are central nervous system tissue. The central nervous system undergoes progressive significant infarction after 12–15′ of non-perfusion [5, 27, 28]. Brain tissue is particularly vulnerable with neuronal death reported to begin after only 5 min of ischemia [29]. Patients undergoing temporary arterial occlusion during intracranial aneurysm surgery for more than 14 min are prone to secondary stroke [30] and a subsequent study found less than 10 min of occlusion generally safe [20]. New neurological deficit from temporary arterial clipping during aneurysm surgery was reported in 30.7% in cases with less than 8 min of clipping but 80.9% with clipping over 8 min [21]. Retinal tissue oxygen consumption per gram is even higher than brain which is one of the most energy consuming organs and it has been suggested the retina is equally or even more vulnerable to oxygen deprivation than brain [31–37]. Cerebral oxygen consumption is about 3.5 ml/100 g/min compared to about 3.8 ml/100 g/min for inner cat retina and between 8 and 13 ml/100 g/min for human retina, the highest in the body [38–41].

Third, oxygenation of the ganglion cells from the vitreous or choroid is clinically insufficient when breathing room air and cannot compensate for complete CRAO by prolonging inner retinal survival time [42, 43].

Consequently, it is most likely that complete CRAO in the human will cause progressive, significant, irreversible retinal ganglion cell death after 12–15 min.

## Discussion

The time to inner retinal infarction from complete CRAO has clinical treatment implications for emergency treatment as well as medical-legal significance.

- *Time to emergency CRAO treatment – the window of opportunity*

If complete CRAO is confirmed by observation of segmentation and stagnation of retinal arterial blood flow for longer than 15 min on successive fundus examinations, treatment is likely to be futile because of inner retinal infarction. However, it is not possible to be certain of the prior duration of a complete CRAO observed on a single fundus examination and a variable degree of reperfusion may occur at any time and intermittently. Even a cherry red spot is not a reliable sign of complete occlusion with retinal infarction because it may occur from inner retinal ischemia without infarction [44]. CRAO is most often incomplete or complete but temporary with subsequent intermittent or permanent degrees of reperfusion resulting from transient arterial spasm or break up of embolus or thrombus [24]. Residual circulation is present demonstrated on fluorescein angiography in most CRAO patients and even a modest level of perfusion may prolong inner retinal survival [45]. Consequently, patients presenting with CRAO usually are offered treatment even if the time from onset of occlusion is believed to be many hours when there is uncertainty regarding completeness and duration of the occlusion. Patients with incomplete occlusions are more likely to benefit from therapy [45] although, unfortunately, no strong evidence based treatment has been shown to be effective for CRAO [1, 3, 6, 9, 46].

Treatments for CRAO include:

- hyperbaric oxygen [47, 48].
- anticoagulation [1, 3].
- vasodilation by retrobulbar injection tolazoline or local anesthetic, sublingual isosorbide dinitrate, oral pentoxifylline, breathing elevated levels of carbon dioxide [1, 3, 7, 49, 50].
- topical ocular hypotensive agents, intravenous mannitol and acetazolamide to decrease extramural arterial tissue pressure by lowering IOP [1, 3, 49].
- paracentesis with release of aqueous from the anterior chamber to lower IOP and restore perfusion or dislodge CRA embolus, an effective method of acute dramatic IOP lowering [1, 50–53].
- ocular massage to lower IOP or dislodge CRA embolus or thrombus [1, 3, 6, 49, 54].
- fibrnolyitic therapy, intravenous or selective intra-arterial thrombolysis most oftenwith tissue plasminogen activator (tPA), popular in some centers but with complications reported in up to 37% of cases [3, 55–67]. Recently, a small uncontrolled series using tPA by intra-retinal arterial micro-cannulation has been reported with limited success [68].

CRAO treatments commonly offered at present are listed in Table 1; however, it is important to note that there is no generally accepted consensus with respect to CRAO treatment and treatment offered at different locations varies greatly according to local protocols and physician experience.

**Table 1:** Common Emergency CRAO Treatment Options

Treatment	Action
Sublingual isosorbide dinitrate	Vasodilation
Oral pentoxifylline	Vasodilation
Carbogen inhalation	Cerebral vasodilation and increased oxygen
Retrobulbar alpha adenergic blocker (tolazoline) or local anesthetic	Vasodilation
Hyperbaric oxygen	Increased oxygen
Ocular massage	Attempt to dislodge emboli or thrombus
Systemic anticoagulation	Limit thrombosis
Intravenous acetazolamine and mannitol	Reduce IOP to increase perfusion pressure
Aqueous humor removal by paracentesis	Reduce IOP to increase perfusion pressure
Thrombolysis, intra-arterial or systemic	Dissolve thrombus

After ophthalmic examination and treatment of retinal stroke from CRAO, further urgent evaluation is indicated to search for a cause of occlusion or the source of a visible embolus because CRAO patients are at elevated risk for other cerebral or myocardial infarction [1, 3, 6].

b) *Medical-legal implications*

Retinal survival time after complete CRAO may have important medical legal implications when a treating doctor is alleged not to have diagnosed or treated in a timely fashion, particularly when CRAO is iatrogenic, for example the result of acute retrobulbar hemorrhage following retrobulbar injection, blepharoplasty, orbital surgery, sinus surgery entering the orbit, and facial injections causing CRA embolization. In such cases, it is very important to recognize that there is no generally accepted, effective treatment for CRAO and also that inner retinal infarction occurs rapidly, within about 15 min if CRAO is complete.

Combined CRAO and choroidal occlusion, sometimes iatrogenic, may be caused by acute, severe, intraocular pressure (IOP) elevation causing infarction of both inner and outer retina as well as optic nerve head with complete permanent loss of retinal function if occlusion is sufficiently prolonged. Such acute IOP elevation has two typical mechanisms:

1. Internal – severe IOP elevation can arise from acute internal ocular volume increase from intraocular injection or intraocular gas expansion following intraocular surgery resulting from too high gas concentration, nitrous oxide anesthesia, or reduced atmospheric pressure during flight or high altitude mountain ascent [69–71].
2. External – acute ocular pressure elevation can result from external pressure on the globe, for example by a head rest during prone general anesthesia, pre-operative ocular compression for intraocular surgery, acute retrobulbar hemorrhage, orbital infection with severe edema, acute large scleral buckle, orbital emphysema, as well as from a variety of objects pressing on the eye [72–82].

Retinal infarction in cases of combined CRAO and choroidal vascular occlusion from acute, highly elevated IOP will occur after only 12–15′ of complete CRAO. Consequently, the eyes of a prone patient positioned on a head rest during general anesthesia should be checked at intervals of 15 min or less.

## Conclusion

Complete CRAO of more than 12–15 min duration is likely to be sufficient to cause stroke of the retinal ganglion cells which form the optic nerve. This may be one reason why no strong evidence based therapy exists for CRAO. If multiple serial fundus examinations show no retinal arterial flow as evidenced by segmentation and stagnation of the arterial blood columns for more than about 15′, treatment is likely to be futile. Otherwise, emergency treatment is offered after much longer intervals in most cases because it is usually not possible to be sure that CRAO has been complete from the onset of symptoms to the time of diagnosis. Retinal stroke from CRAO requires further urgent evaluation, after ophthalmic examination and treatment, to search for a cause of occlusion because CRAO patients are at elevated risk for other cerebral or myocardial infarction. To try to avoid CRAO from inadvertent ocular compression by a headrest during prone anesthesia, the eyes should be checked at intervals of less than 15′.

## Abbreviations

CRA: Central retinal artery
CRAO: Central retinal artery occlusion
IOP: Intraocular pressure
